# Causal relationship between air pollution and infections: a two-sample Mendelian randomization study

**DOI:** 10.3389/fpubh.2024.1409640

**Published:** 2024-08-01

**Authors:** Shengyi Yang, Tong Tong, Hong Wang, Zhenwei Li, Mengmeng Wang, Kaiwen Ni

**Affiliations:** Department of Infection Control, Second Affiliated Hospital of Zhejiang University School of Medicine, Hangzhou, Zhejiang, China

**Keywords:** air pollution, infections, pneumonia, Mendelian randomization, casual effect

## Abstract

**Background:**

Traditional observational studies exploring the association between air pollution and infections have been limited by small sample sizes and potential confounding factors. To address these limitations, we applied Mendelian randomization (MR) to investigate the potential causal relationships between particulate matter (PM2.5, PM2.5–10, and PM10), nitrogen dioxide, and nitrogen oxide and the risks of infections.

**Methods:**

Single nucleotide polymorphisms (SNPs) related to air pollution were selected from the genome-wide association study (GWAS) of the UK Biobank. Publicly available summary data for infections were obtained from the FinnGen Biobank and the COVID-19 Host Genetics Initiative. The inverse variance weighted (IVW) meta-analysis was used as the primary method for obtaining the Mendelian randomization (MR) estimates. Complementary analyses were performed using the weighted median method, MR-Egger method, and MR Pleiotropy Residual Sum and Outlier (MR-PRESSO) test.

**Results:**

The fixed-effect IVW estimate showed that PM2.5, PM2.5–10 and Nitrogen oxides were suggestively associated with COVID-19 [for PM2.5: IVW (fe): OR 3.573(1.218,5.288), P_IVW(fe)_ = 0.021; for PM2.5–10: IVW (fe): OR 2.940(1.385,6.239), P_IVW(fe)_ = 0.005; for Nitrogen oxides, IVW (fe): OR 1.898(1.318,2.472), P_IVW(fe)_ = 0.010]. PM2.5, PM2.5–10, PM10, and Nitrogen oxides were suggestively associated with bacterial pneumonia [for PM2.5: IVW(fe): OR 1.720 (1.007, 2.937), P_IVW(fe)_ = 0.047; for PM2.5–10: IVW(fe): OR 1.752 (1.111, 2.767), P _IVW(fe)_ = 0.016; for PM10: IVW(fe): OR 2.097 (1.045, 4.208), P_IVW(fe)_ = 0.037; for Nitrogen oxides, IVW(fe): OR 3.907 (1.209, 5.987), P_IVW(fe)_ = 0.023]. Furthermore, Nitrogen dioxide was suggestively associated with the risk of acute upper respiratory infections, while all air pollution were not associated with intestinal infections.

**Conclusions:**

Our results support a role of related air pollution in the Corona Virus Disease 2019, bacterial pneumonia and acute upper respiratory infections. More work is need for policy formulation to reduce the air pollution and the emission of toxic and of harmful gas.

## 1 Background

Bacterial pneumonia, Corona Virus Disease 2019 (COVID-19), acute upper respiratory infections, and intestinal infections are common causes of hospital admission and important contributors to death (1). Among respiratory infectious diseases, lower respiratory tract infections (LRTIs) and pneumonia, in particular, rank in highest in terms of mortality (2). Several factors are implicated in heightening the risk of infection, including age, vaccination coverage, antibiotic therapy, seasonal fluctuations, and air pollution (3).

Notably, the persistently rising levels of air pollution worldwide have yielded dire consequences, leading to an alarming number of premature deaths. This increase in pollution is linked to a higher incidence of infections, especially respiratory infections (4, 5). Consequently, the role of respiratory infections as a driver of human mortality has assumed a position of paramount concern and it is now more crucial than ever to gain a comprehensive understanding of the intricate interplay between air pollution and infections. Particulate matter (PM) serves as a key indicator of air pollution, resulting from a variety of natural and human activities (6). Traditional observational studies have explored the association between PM and infections. Evidence from China by Yongjian Zhu indicated significantly positive associations of PM2.5, PM10, and Nitrogen dioxide (NO_2)_ over the past 2 weeks with newly confirmed COVID-19 cases (7). However, this study did not include gender- or age-specific cases. Another study in Italy found that long-term air quality data with cases of COVID-19 in up to 71 Italian provinces, further indicating that chronic exposure to air pollution could facilitate virus spread (8). A systematic review with 15 studies by Chiara Copat indicated PM2.5 and NO_2_ are more closely correlated to COVID-19 than PM10 (9). Notably, these studies included limited sample sizes with potential confounders.

Investigating the causal association between air pollution and the risk of infections is challenging due to reverse causation and confounding. Mendelian randomization (MR) has emerged as a potent method for determining causation between risk factors and diseases by using genetic variants as instrument variables (IVs) (10). MR analysis can largely overcome the confounders with random assignment of an individual's genetic variants at conception. Moreover, it minimizes the risk of reverse causation, since the presence of a disease cannot affect individuals' genotypes (11).

In our study, we applied a two-sample MR analysis to explore the potential causal association between air pollution (including PM2.5, PM2.5–10, PM10, nitrogen dioxide, and nitrogen oxides) and risk of infections (intestinal infections, acute upper respiratory infections, bacterial pneumonia, and Corona Virus Disease 2019) using the summary statistics derived from the publicly available GWAS data.

## 2 Materials and methods

### 2.1 Study design

**Figure 1:**
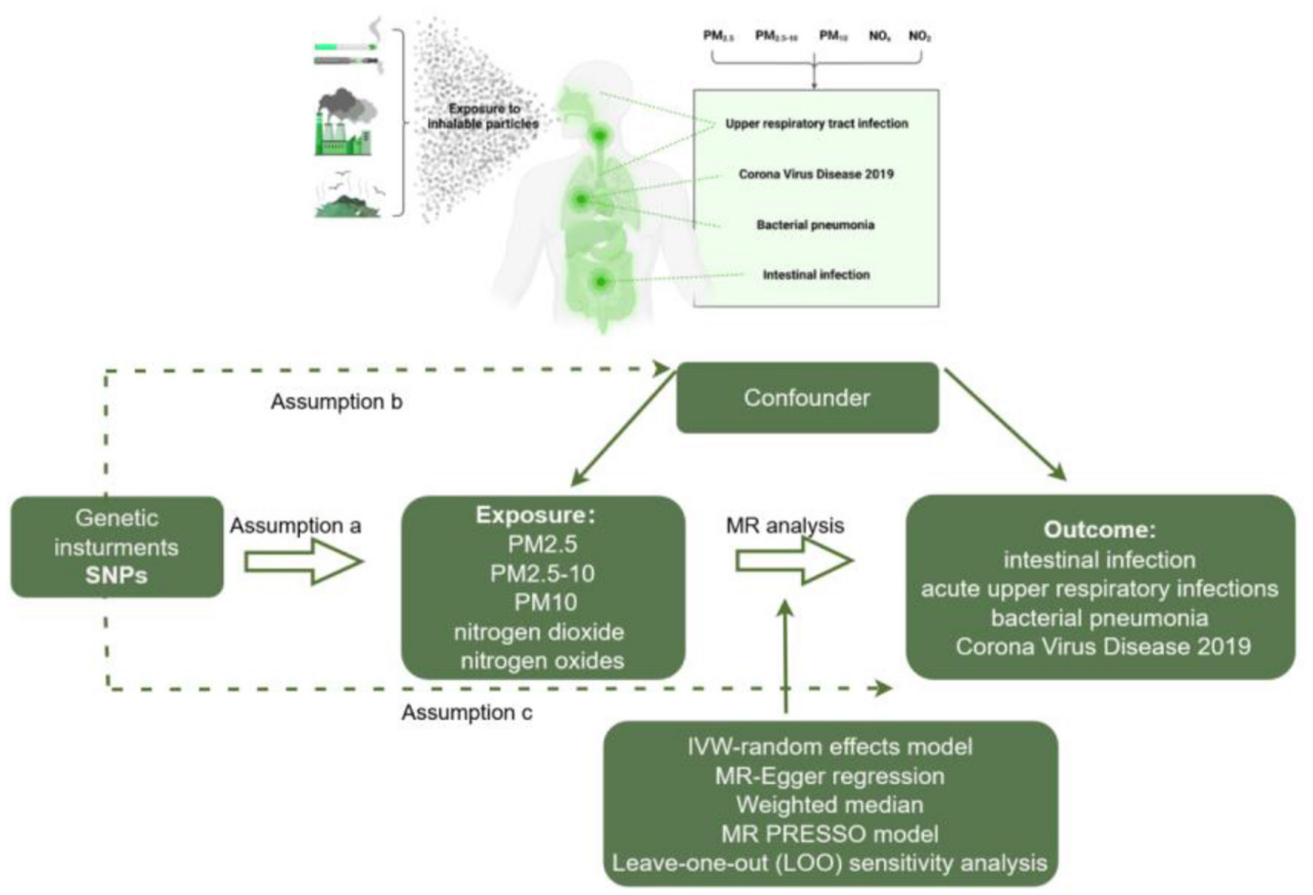
Workflow of two-sample MR for causal effect between air pollution and infection risks. PM, Particulate matter.

This study is reported according to the STROBE-MR guidelines (12). Our study is based on the Mendelian randomization design to explore the causal relationship between Air pollution and infections using publicly available summary datasets from two genome-wide association studies. In this study, air pollution indicators (PM2.5, PM2.5–10, PM10, nitrogen dioxide, and nitrogen oxides) were selected as exposures, while various infections (Intestinal infections, acute upper respiratory infections, bacterial pneumonia, and Corona Virus Disease 2019) served as outcomes. The design of this MR study is presented in Figure 1.

### 2.2 Data retrieval for MR analyses

We collected summary data on single nucleotide polymorphism (SNP)–phenotype associations from different Genome-wide Association Studies (GWAS). Publicly available summary data for PM2.5, PM2.5–10, PM10, nitrogen dioxide, and nitrogen oxides were obtained from UK Biobank, including more than 400,000 participants with European ancestors (13). UK Biobank is a large-scale biomedical database and research resource, containing in-depth genetic and health information from half a million UK participants (https://www.ukbiobank.ac.uk/). Air pollution-related indicators were measured by land use regression (LUR) models (14).

Publicly available summary data for Corona Virus Disease 2019 was from COVID-19 Host Genetics Initiative including 1,887,658 European participants (15). One hundred and five studies have joined the initiative, and participation is still expanding. The majority of studies are conducted in Europe (55%) and the US (28%) (15). Summary data for bacterial pneumonia, acute upper respiratory infections and intestinal infections were both from FinnGen Biobank with European ancestors (215,268 for Bacterial pneumonia, 218,792 for acute upper respiratory infections, 200,006 for intestinal infections). The GWASs conducted on the FinnGen dataset were analyzed using SAIGE and were adjusted for sex, age, first ten principal components, and genotyping batch (16). The detailed information was presented in Table 1.

**Table 1:** Details of the genome-wide association studies included in this Mendelian randomization analysis.

**Exposures/outcomes**		**Consortium**	**Ethnicity**	**Participants**	**No. of SNPs**	**Sex**
Air pollution	Particulate matter (PM) 2.5 um	UK Biobank	European	423,796	9,851,867	Males and Females
	Particulate matter (PM) 2.5–10 um	UK Biobank	European	423,796	9,851,867	Males and Females
	Particulate matter (PM) 10 um	UK Biobank	European	455,314	9,851,867	Males and Females
	Nitrogen dioxide	UK Biobank	European	456,380	9,851,867	Males and Females
	Nitrogen oxides	UK Biobank	European	456,380	9,851,867	Males and Females
Infections	Intestinal infections	FinnGen Biobank	European	200,006	16,380,395	Males and Females
	Acute upper respiratory infections	FinnGen Biobank	European	218,792	16,380,466	Males and Females
	Bacterial pneumonia	FinnGen Biobank	European	215,268	16,380,460	Males and Females
	Corona Virus Disease 2019	COVID-19 Host Genetics Initiative	European	1,887,658	8,107,040	Males and Females

COVID-19, Corona Virus Disease 2019; PM, Particulate matter.

### 2.3 Genetic instrumental variables selection

All genetic variants reaching genome-wide significance (*P* < 5 × 10^−8^) were selected as instruments for the MR analysis. To minimize the potential for weak instrumental variable bias, we employed a screening criterion of *P* < 5 × 10^−6^ for linear regression of each genetic variant on the respective risk factor (17–19).

Additionally, we lowered the genome-wide significance threshold of the PM2.5–10 to *P* < 5 × 10^−6^ to select enough SNPs as IVs associated with this significance level (17–19). The corresponding linkage disequilibrium was tested to confirm that there were any SNPs in a linkage disequilibrium state and the SNPs were independent by pruning SNPs within a 10,000 kb window with an R^2^ < 0.001 threshold (20). Furthermore, we identified SNPs associated with potential confounders of the outcomes. In this study, obesity, pregnancy, cardiovascular disease, and Parkinson's disease were considered confounding factors when COVID-19 was identified as the outcome (21–24). BMI, alcohol intake, asthma and coronary artery disease were considered confounding factors when Bacterial pneumonia and acute upper respiratory infections were identified as the outcomes (25, 26). Furthermore, Proton pump inhibitor (PPI) was considered confounding factors when Intestinal infections were identified as the outcome (http://www.phenoscanner.medschl.cam.ac.uk/) (27–29). SNP harmonization was conducted to correct the orientation of the alleles. We used the F statistic and R^2^ to evaluate the strength of the association between SNP and exposure and were also conducted to further assess weak instrument (30, 31). A strong correlation between SNP and exposure with sufficient statistical power was confirmed when the F statistic is >10 (Table 2).

**Table 2:** The F statistic, heterogeneity and horizontal pleiotropy test between the exposures and outcomes of this MR.

**Exposures and outcomes**	**PM2.5**	**PM2.5–10**	**PM10**	**Nitrogen oxides**
Corona Virus Disease 2019	•^a^•^b^•^c^	•••	•••	•••
Bacterial pneumonia	•••	•••	•••	•••
Acute upper respiratory infections	•••	•••	•••	•••
Intestinal infections	• ° •	•••	• ° •	• ° •

a, •means F statistic > 10; b, •means heterogeneity existence; °means no heterogeneity existence; c, •means horizontal pleiotropy existence.PM, particular matter.

### 2.4 Two-sample MR analysis

Two-sample MR analysis were performed to explore the potential causal associations between air pollution and infections in two populations, respectively. For a genetic variant to be qualified as a valid instrument for causal inference in a MR study, it must meet three core assumptions (32):

a. The genetic variant must be truly associated with the exposure;

b. The genetic variant should not be associated with confounders of the exposure-outcome relationship; and

c. The genetic variant should only be related to the outcome of interest through the exposure under study.

The inverse variance weighted (IVW) meta-analysis was used as the main method for obtaining the MR estimate (33). Complementary analyses were performed using the weighted median method (34) and MR-egger method (34). Cochran's Q test and I^2^ was applied to assess heterogeneity between individual genetic variants estimates, by which random-effects model or fixed-effects model of IVW was determined (35).

Furthermore, we have performed another one-sample MR analysis as Replicative analysis. Summary statistics of air pollution and COVID-19 were all obtained from the (GWAS) UK Biobank.

### 2.5 Sensitivity analysis

To examine the possibility of violation of the main MR assumptions due to directional pleiotropy, the MR-Egger test for directional pleiotropy was performed (34). In this test, the intercept estimates the average pleiotropic effect across the genetic variants (36). Additionally, the MR pleiotropy residual sum and outlier test (MR-PRESSO) was performed to detect and correct the effects from outliers (37). To further assess the independent potential of each IV, a leave-one-out (LOO) sensitivity analysis was also performed. A forest plot was generated to evaluate the robustness of our results.

### 2.6 Statistical analysis

All analyses were performed using the package “Two-Sample-MR” (version 0.5.6) and “MR-PRESSO” (version 1.0) in R (version 4.0.5). A two-sided *P* value of < 0.05 was considered a potential causal relationship. To account for multiple testing in our primary analyses, a Bonferroni corrected threshold of P was applied.

## 3 Results

### 3.1 Genetic instrumental variables selected

After genetic Instrumental Variables Selections, the detailed information of the IVs was presented in Supplementary Table 1. F statistics for every instrument-exposure association were >10 in our study, demonstrating the small possibility of weak instrumental variable bias. Furthermore, the excluded SNPs associated with confounder risks were presented in Supplementary Tables 1, 2.

### 3.2 PM2.5, PM2.5–10, and nitrogen oxides will increase the risk of Corona Virus Disease 2019

**Figure 2:**
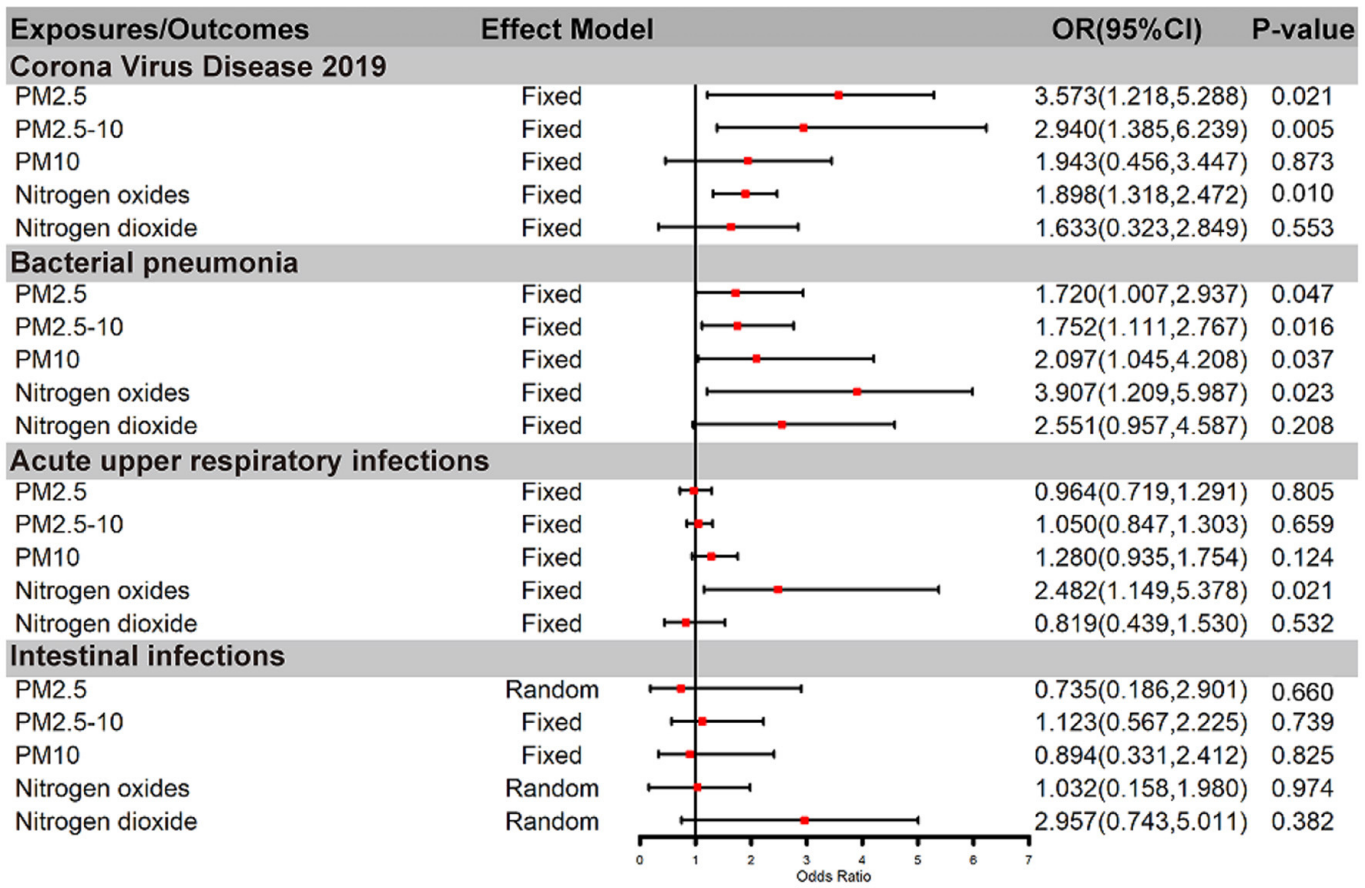
Causal relationship between air pollution and infections by IVW method. OR, odds ratio; PM, Particulate matter.

**Figure 3:**
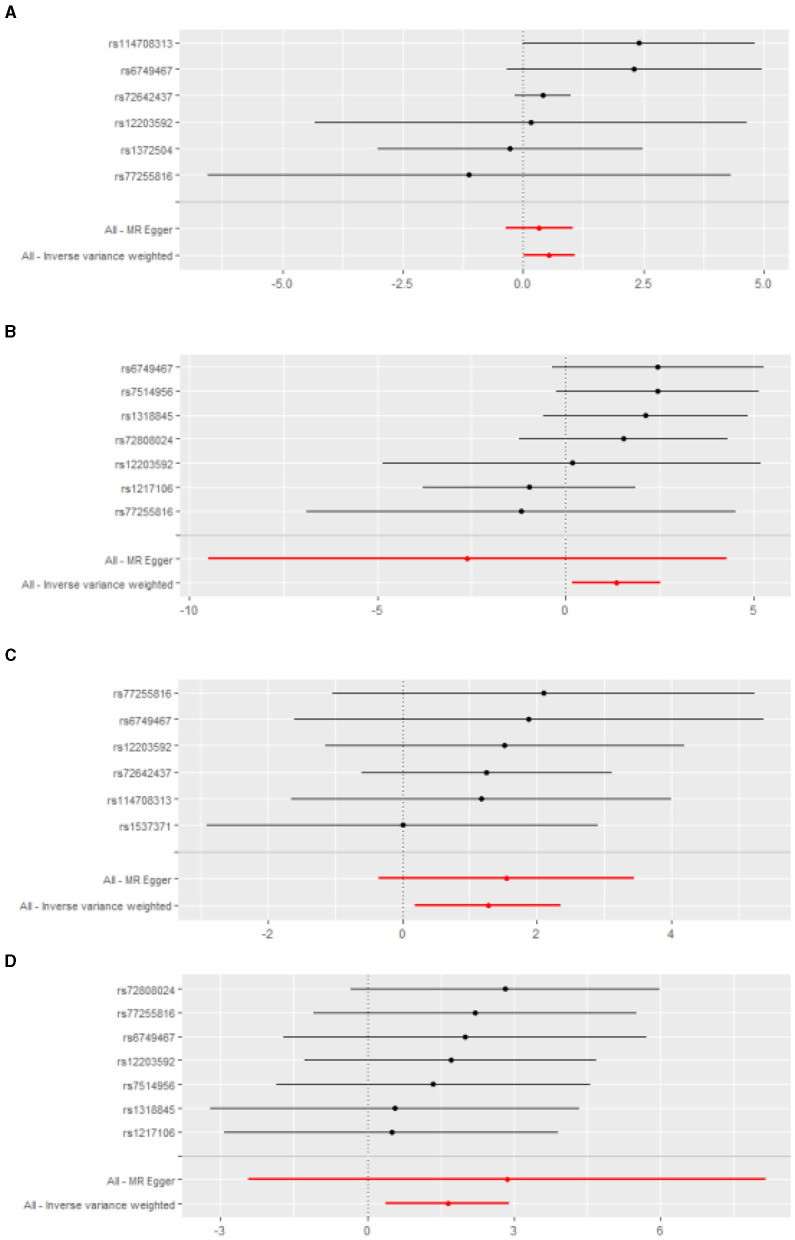
The forest plots for the effect of air pollution on the infections. **(A)** PM2.5 and bacterial pheumonia; **(B)** Nitrogen oxides and bacterial pheumonia; **(C)** PM2.5 and COVID-19; **(D)** Nitrogen oxides and COVID-19.

After genetic instrumental variables selected, 6, 21, 20, 7, and 4 SNPs for PM2.5, PM2.5–10, PM10, nitrogen dioxide, and nitrogen oxides were identified after removal of chained unbalanced IVs. Table 3 reported the MR estimated for the association between air pollution and Corona Virus Disease 2019. The fixed-effect IVW estimate showed that PM2.5 and PM2.5–10 were significantly associated with Corona Virus Disease 2019 (for PM2.5: P_IVW(fe)_ = 0.021; for PM2.5–10, P_IVW(fe)_ = 0.005). However, we did not observe evidence of causal association in Bonferroni correction. To ensure the robustness of our results, MR-PRESSO was also conducted which showed the similar results (for PM2.5: P_MR − PRESSO_ = 0.037; for PM2.5–10: P_MR − PRESSO_ = 0.013). Furthermore, The IVW method showed that Nitrogen oxides was significantly associated with Corona Virus Disease 2019 (P_IVW(fe)_ = 0.010), while PM10 and Nitrogen dioxide were not the risk factor of it (for PM10: P_IVW(fe)_ = 0.873; for Nitrogen dioxide: P _IVW(fe)_ = 0.533). Figure 2 also presented the MR results by IVW estimate. And we also found that the genetically predicted PM2.5 um was positively associated with increased risk of COVID-19 with IVW method by replicative analysis (Supplementary Tables 4, 5). There was no evidence of heterogeneity or directional pleiotropy for the analysis of air pollution on COVID-19 (Table 2, Supplementary Table 6). The forest plots were displayed in Figure 3. The Leave-one-out sensitivity analysis of MR estimate were presented in Supplementary Figures 1–3.

**Table 3:** The association between air pollution and infections.

**Exposure**	**Outcome**	**MR**				
		**nSNP**	**Methods**	**Beta**	**OR (95%CI)**	***P*** **-value**
PM2.5	COVID-19	6	IVW(re)	1.273	3.572 (1.211, 5.213)	0.021
		6	IVW(fe)	1.273	3.573 (1.218, 5.288)	0.021
		6	MR-PRESSO	0.986	2.680 (1.132, 6.350)	0.037
		6	Weighted median	1.302	3.677 (0.954, 6.597)	0.058
		6	MR-Egger	0.432	2.706 (0.701, 4.622)	0.186
PM2.5–10	COVID-19	21	IVW(re)	1.078	2.939 (1.350, 6.398)	0.007
		21	IVW(fe)	1.078	2.940 (1.385, 6.239)	0.005
		21	MR-PRESSO	1.078	2.938 (1.350, 6.399)	0.013
		21	Weighted median	0.819	2.269 (0.697, 7.387)	0.173
		21	MR-Egger	0.804	2.233 (0.456, 5.776)	0.333
Nitrogen oxides	COVID-19	7	IVW(re)	0.641	1.898 (1.464, 2.311)	0.011
		7	IVW(fe)	0.641	1.898 (1.318, 2.472)	0.01
		7	MR-Egger	0.851	2.342 (0.739, 2.673)	0.339
		7	MR-PRESSO	0.641	1.898 (1.034, 3.485)	0.002
		7	Weighted median	0.702	2.342 (1.175, 3.502)	0.036
PM2.5	Bacterial pneumonia	6	IVW(re)	0.542	1.720 (1.008, 2.937)	0.046
		6	IVW(fe)	0.542	1.720 (1.007, 2.937)	0.047
		6	MR-Egger	0.329	1.389 (0.693, 2.781)	0.406
		6	MR-PRESSO	0.490	1.632 (1.010, 2.237)	0.039
		6	Weighted median	0.455	1.576 (0.867, 2.863)	0.136
PM2.5–10	Bacterial pneumonia	21	IVW(re)	0.642	1.899 (1.228, 2.938)	0.004
		21	IVW(fe)	0.561	1.752 (1.111, 2.767)	0.016
		21	MR-Egger	0.396	1.486 (0.775, 2.849)	0.248
		20	MR-PRESSO	0.543	1.721 (1.042, 0.843)	0.027
		21	Weighted median	0.466	1.594 (0.824, 3.084)	0.166
PM10	Bacterial pneumonia	17	IVW(re)	0.741	1.097 (1.045, 2.147)	0.037
		17	IVW(fe)	0.741	2.097 (1.045, 4.208)	0.037
		17	MR-Egger	0.258	1.295 (0.242, 6.930)	0.767
		17	MR-PRESSO	0.387	1.473 (0.694, 3.125)	0.327
		17	Weighted median	0.403	1.450 (0.581, 3.854)	0.404
Nitrogen oxides	Bacterial pneumonia	7	IVW(re)	1.362	3.907 (2.224, 5.611)	0.023
		7	IVW(fe)	1.362	3.907 (1.209, 5.987)	0.023
		7	MR-Egger	−2.610	0.073 (0.000, 1.126)	0.491
		7	MR-PRESSO	1.362	3.904 (3.540, 4.306)	0.048
		7	Weighted median	1.441	4.225 (1.156, 7.326)	0.012
Nitrogen dioxide	Acute upper respiratory infections	5	IVW(re)	0.912	2.486 (1.149, 5.378)	0.021
		5	IVW(fe)	0.912	2.482 (1.149, 5.378)	0.021
		5	MR-Egger	0.502	1.652 (0.390, 6.990)	0.245
		5	MR-PRESSO	0.112	1.105 (0.595, 2.053)	0.044
		5	Weighted median	0.931	2.537 (0.933, 6.894)	0.068

SNP, single nucleotide polymorphism; MR, Mendelian randomization; PM, particulate matter; COVID-19, Corona Virus Disease 2019; IVW, inverse variance weighted; fe, fixed effect; re, random effect; MR-PRESSO, MR pleiotropy residual sum and outlier test; OR, odds ratio; CI, confidence interval.

### 3.3 PM2.5, PM2.5–10, PM10, and Nitrogen oxides will increase the risk of bacterial pneumonia

After genetic instrumental variables selected, 6, 21, 17, 7 and 5 SNPs for PM2.5, PM2.5–10, PM10, nitrogen dioxide, and nitrogen oxides were identified after removal of chained unbalanced IVs. The fixed-effect IVW estimate showed that PM2.5, PM2.5–10, PM10, and Nitrogen oxides were significantly associated with bacterial pneumonia (for PM2.5: P_IVW(fe)_ = 0.047; for PM2.5–10: P _IVW(fe)_ = 0.016; for PM10: P _IVW(fe)_ = 0.037; for Nitrogen oxides: P _IVW(fe)_ = 0.023, Table 3, Figure 2, Supplementary Table 3). However, we did not observe evidence of causal association in Bonferroni correction. In addition, the IVW method showed that Nitrogen dioxide was not significantly associated with bacterial pneumonia (P _IVW(fe)_ = 0.208). The results were consistent in complementary analyses (P_MR − Egger_ = 0.858, P_Weightedmedian_ = 0.518, P_MR − PRESSO_ = 0.518). There was no evidence of heterogeneity or directional pleiotropy for the analysis (Table 2, Supplementary Table 6). And the forest plots were displayed in Figure 3.

### 3.4 Air pollution was not associated with the risk of acute upper respiratory infections and Intestinal infections except Nitrogen dioxide

The IVW estimate support that Nitrogen dioxide was associated with the risk of acute upper respiratory infections which were consistent with the result by MR-PRESSO method (P_IVW(fe)_ = 0.021, P_MR − PRESSO_ = 0.044, Table 3, Figure 2, Supplementary Table 3). However, we did not observe evidence of causal association in Bonferroni correction. All method did not support that PM2.5, PM2.5–10, PM10, and nitrogen dioxide were associated with Acute upper respiratory infections and intestinal infections. There was no evidence of heterogeneity or directional pleiotropy for the analysis of air pollution on acute upper respiratory infections (Table 2, Supplementary Table 6). However, Cochran's Q test showed that there was heterogeneity for the analysis of PM2.5, nitrogen oxides, and nitrogen dioxide on intestinal infections. And the forest plots were displayed in Figure 3.

## 4 Discussion

In this two-sample MR study, we found that PM2.5, PM2.5–10, and nitrogen oxides increase the risk of Coronavirus Disease 2019. Furthermore, PM2.5, PM2.5–10, PM10, and nitrogen oxides are associated with an increased risk of bacterial pneumonia. However, our analysis did not reveal a suggestively association between air pollution and the risk of acute upper respiratory infections and intestinal infections, except for nitrogen dioxide.

As mentioned, exposure to PM is associated with upper and lower respiratory tract infections. The COVID-19 pandemic, caused by the novel severe acute respiratory syndrome coronavirus 2 (SARS-CoV-2), has resulted in historic numbers of infections and deaths worldwide over the last 3 years (2022). Consequently, an increasing number of studies have investigated associations between PM and COVID-19 infection in China, Italy, and the USA (7–9, 38). The mechanism of COVID-19 infection related to PM can be summarized as follows. (1) PM may be involved in different life cycle stages of COVID-19, including alteration of SARS-CoV-2 viral receptors and proteases required for entry (e.g., angiotensin-converting enzyme 2 (ACE2) and transmembrane protease serine type 2 (TMPRSS2), proteins and protease critical to SARS-CoV-2 entry into host cells) (39), inhibition of mucosal ciliary clearance, alteration of antiviral interferon production and viral replication (40). (2) PM may impair the immune system. PM-induced pro-inflammatory cytokine production, oxidative stress, and impaired airway immune function may lead to increased susceptibility to respiratory pathogens, which can increase the risk of COVID-19 pneumonia (41). For example, exposure to chemicals in PM damages lung epithelial cells, interferes with tight junctions between epithelial cells, increases permeability of airway and lung epithelial cells, and decreases protection against viral infections (42). Furthermore, substances such as heavy metals and polycyclic aromatic hydrocarbons promote the production of reactive oxygen species in lung cells (40), increasing susceptibility to further oxidative damage due to oxidative stress. Interestingly, our results, consistent with findings by Bontempi (43), revealed a closer correlation between PM2.5 and COVID-19 than with PM10. This may be because PM10 larger than 5 μm is unlikely to reach type II alveolar cells, where the ACE2 receptor for cellular entry is primarily located (44).

The relationship between NO_2_ and COVID-19 has been reported as positive in China, Europe, and the United States (9). However, contrasting evidence suggesting a negative or insignificant association between NO_2_ and COVID-19 also exists (45, 46). The potential effect of NO_2_ exposure remains uncertain. Studies on nitrogen oxides (NO_x_) and COVID-19 are scarce. We demonstrated that there is a significant association between NO_x_ and COVID-19. This may be determined by the next mentioned physicochemical properties of NO_x_ that are not readily soluble in water. In addition, a study by Pfeffer et al. discovered that higher environmental NO_x_ levels were linked to exacerbated viral lung infections, which increases the susceptibility to concurrent bacterial infections and enhances its severity (47).

Pneumonia has been a cause of morbidity and mortality throughout human history (48). Bacterial pneumonia represents the most prevalent manifestation of pneumonia, characterized by inflammation affecting the terminal airways, alveoli, or interstitial spaces because of pathogenic microorganism infections (49). Pathogenetic investigations have indicated Streptococcus pneumoniae as potentially the most widespread bacterial agent responsible for pneumonia on a global scale (50). Over the past few decades, the rise in mortality rates associated with bacterial pneumonia has been attributed, in part, to worsening air quality and the proliferation of environmental hazards. These factors facilitate pathogen transmission, often synergizing with particulate matter (PM) (51). Air pollution poses a significant challenge to public health, exacerbating the potential for pathogen epidemics.

Our study establishes a potential causal relationship between PM2.5, PM2.5–10, PM10, and nitrogen oxides (mainly nitric oxide), all of which exhibit associations with bacterial pneumonia. It is widely acknowledged that respirable particles encompass a complex amalgamation of ions, organic compounds, metals, carbonaceous matter, and other constituents (52). The composition and relative abundance of these components are primarily contingent upon factors such as source origin, climatic conditions, topography, and other environmental variables (53). Consequently, respirable particles may exhibit varying degrees of solubility, ranging from partial solubility to complete insolubility (54). Furthermore, nitrogen oxides (mainly nitric oxide) possess limited solubility and does not undergo reactions with water. These physical properties, to some extent, contribute to the characteristics of these air pollutants in the context of lower respiratory tract infections. Currently, a plethora of epidemiological and mechanistic studies lends support to our conclusion. Notably, a meta-analysis has demonstrated a causal relationship between PM2.5 exposure and acute lower respiratory tract infections (55).

In the case of Chile, respirable particles have been associated with an elevated frequency of emergency room visits among children under the age of 2 experiencing lower respiratory symptoms (56). Conversely, no significant association has been observed between indoor NO_2_ concentrations and the incidence or severity of respiratory illnesses in infants (57). Furthermore, urban PM exposure can promote bacterial adherence to human respiratory epithelial cells by impairing mucus cilia activity in airway mucosa (58, 59), as well as promoting bacterial adherence to human respiratory epithelial cells (60) to enhance bacterial colonization of the host lower respiratory tract.

On the other hand, our findings indicate that only NO_2_ suggestively constitutes a risk factor for upper respiratory tract infections, with its soluble nature dictating the characteristics of this air pollutant, primarily impacting the upper respiratory tract (54). In a study conducted by Arbex et al. (61), it was observed that a mere 10 μg/m^3^ rise in NO_2_ concentration correlated with a 0.63% increase in visits for upper respiratory tract infections. Likewise, two researches conducted in China (62, 63) reported that each 10 μg/m^3^ increment in NO_2_ levels corresponded to a 1.00% increase in emergency room visits for upper respiratory tract infections, along with a substantial 11.27% surge in pediatric emergency room visits related to upper respiratory tract infections. However, it is important to note that while NO_2_ may not directly enter the pulmonary system, its potent oxidizing properties trigger an inflammatory response, subsequently activating the body's immune system and affecting the entire respiratory system (64).

The SNP rs12203592, located in the IRF4 gene intron, is linked to pigmentation traits, hematological traits, squamous cell carcinoma, and smoking cessation. A multi-ethnic GWAS identified it as a novel lung cancer locus. It also increases the risk of invasive aspergillosis post-hematopoietic stem cell transplantation by modulating IRF4 mRNA expression and immune responses (65, 66). Another SNP, rs1537371, in the CDKN2B-AS1 gene, is linked to cardiovascular diseases like coronary artery disease (67, 68). SNPs in the 9p21 region, such as rs1537371 and rs1333049, are significantly associated with an increased risk of cardiovascular disease (69). This study finds rs1537371 and rs12203592 significantly associated with air pollution-related COVID-19 and bacterial pneumonia risks, suggesting pleiotropic effects (70).

These findings offer potential causal relationship between air pollutant exposure and the incidence of respiratory infections. Notably, it is of concern that a staggering 91% of the world's population resides in regions where air pollution exceeds the limits recommended by the World Health Organization (WHO) (source: https://www.who.int/news-room/fact-sheets/detail/ambient-(outdoor)-air-quality-and-health). Therefore, taking steps to curtail air pollution has the potential to yield significant reductions in the global burden of respiratory infectious diseases. Consequently, our data underscore the importance of advocating for worldwide initiatives aimed at reducing air pollution and transitioning to more sustainable energy sources that yield cleaner air.

There are some strengths should be mentioned as following: Firstly, this is the first MR analysis to examine the casual effect of air pollution on infections using large-scale GWAS data. Comparing to the observational studies, MR analysis can largely overcome the confounders with random assignment of an individual's genetic variants at conception. Moreover, the risk of reverse causation could also be minimized. Secondly, we conducted sensitivity analysis both with and without outliers to detect any coincidental effects, and all models exhibited no directional pleiotropy. Finally, F statistics were consistently >10, indicating a robust correlation between SNPs and exposures with the sufficient statistical power and minimizing the possibility of weak instrumental variable bias.

There are some shortcomings in this MR study. Firstly, there was heterogeneity among our results. Due to the GWAS data, any potential non-linear relationships or stratification effects which varies by health status, age or sex cannot be examined which may be the resource of heterogeneity. Secondly, our study could not rule out the effect of canalization (i.e., dilution of the gene-exposure association) and thus the estimate might be inflated (71). Thirdly, the association between air pollution and different infections subtypes was not explored. Additionally, instances where air pollution levels exceed the standard cannot be entirely discounted, as such occurrences may introduce confounding factors that could bias our MR estimates. Finally, our datasets included the European populations, limiting the applicability of our results to non-European populations. Further studies are needed to verify the applicability of these results in other populations and ethnicities.

## 5 Conclusion

In conclusion, we demonstrated a causal association between PM2.5, PM2.5–10, and nitrogen oxides on Corona Virus Disease 2019. Additionally, PM2.5, PM2.5–10, PM10, and Nitrogen oxides will increase the risk of bacterial pneumonia. However, air pollution, except for nitrogen dioxide, was not associated with the risk of acute upper respiratory infections. Overall, more work is needed for policy formulation to reduce air pollution and the emission of toxic and harmful gases.

## Data availability statement

The original contributions presented in the study are included in the article/Supplementary material, further inquiries can be directed to the corresponding author.

## Author contributions

SY: Conceptualization, Data curation, Formal analysis, Investigation, Methodology, Project administration, Resources, Software, Supervision, Validation, Visualization, Writing – original draft, Writing – review & editing. TT: Conceptualization, Data curation, Formal analysis, Investigation, Project administration, Resources, Supervision, Validation, Writing – original draft, Writing – review & editing. HW: Conceptualization, Data curation, Formal analysis, Investigation, Project administration, Resources, Supervision, Validation, Writing – original draft, Writing – review & editing. ZL: Data curation, Resources, Supervision, Validation, Writing – review & editing. MW: Data curation, Resources, Supervision, Validation, Writing – review & editing. KN: Conceptualization, Data curation, Formal analysis, Investigation, Methodology, Project administration, Resources, Software, Supervision, Validation, Visualization, Writing – review & editing.
