# Supplementary figures and images for: Causal relationship between air pollution and infections: a two-sample Mendelian randomization study
Source: Front Public Health. 2024 Aug 1;12:1409640. doi: 10.3389/fpubh.2024.1409640 (PMC11324489; doi:10.3389/fpubh.2024.1409640)

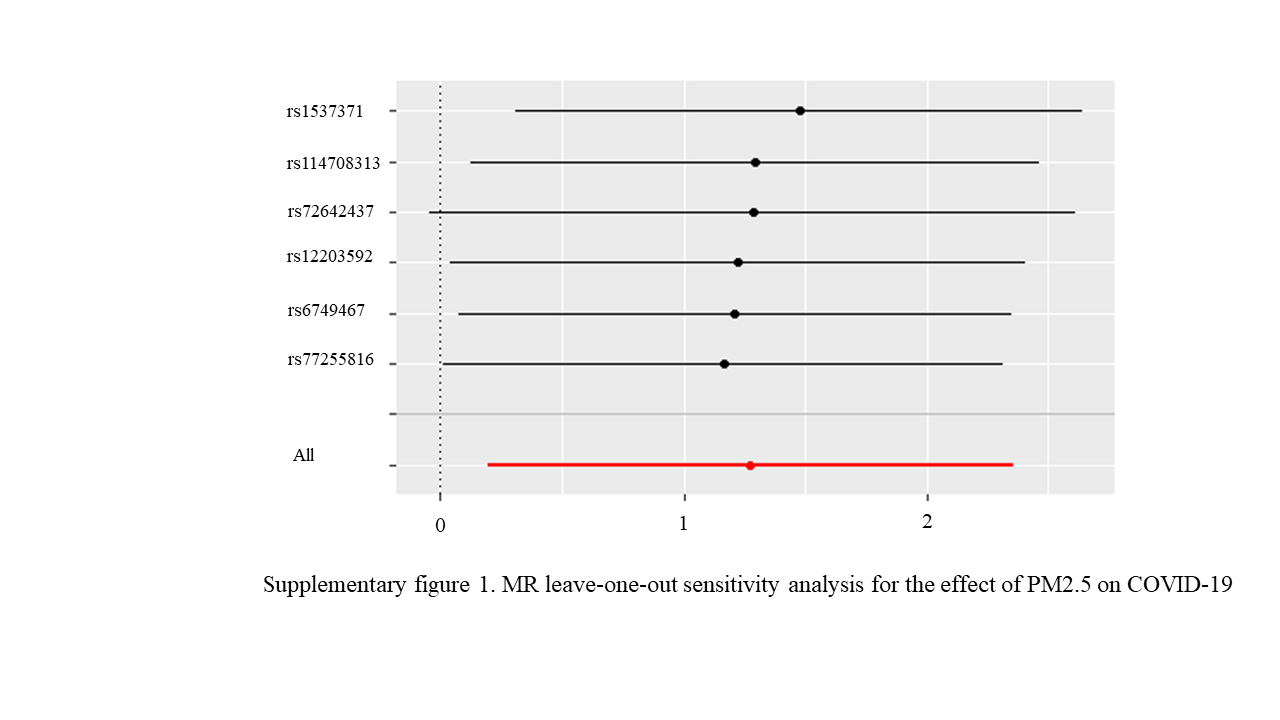

Supplement: Supplementary file 2 [file Image_1.TIF]

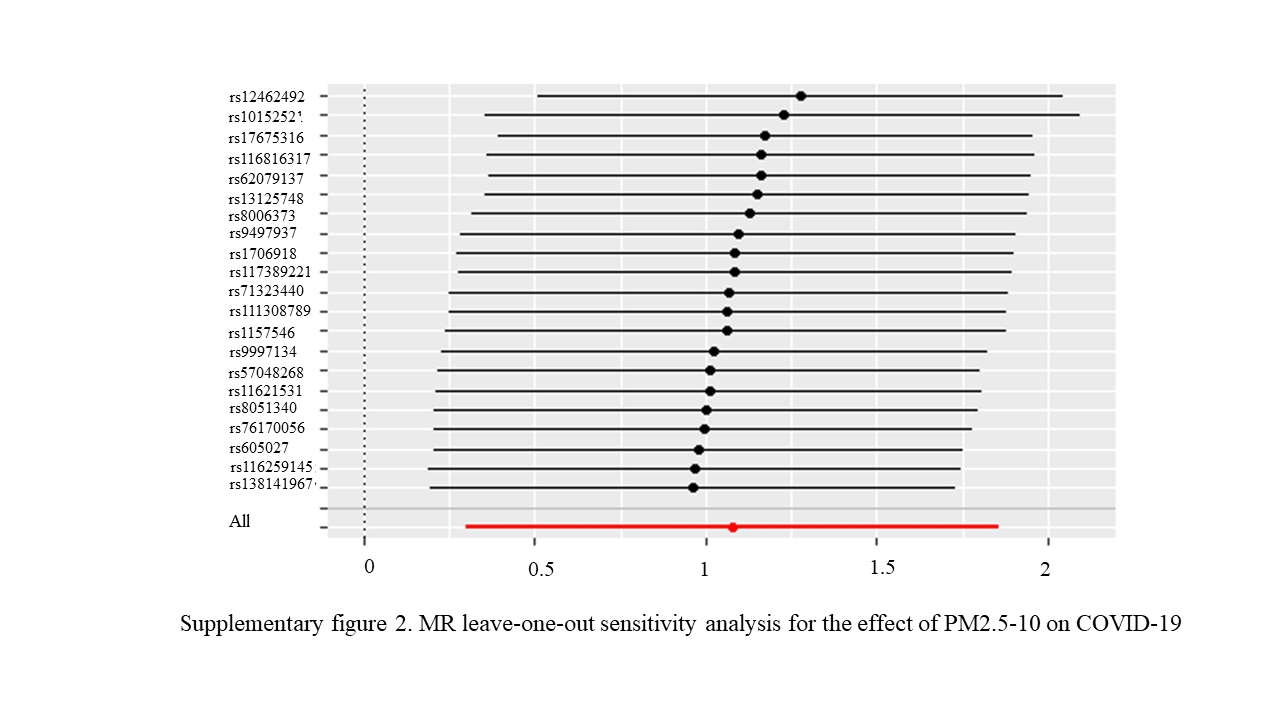

Supplement: Supplementary file 3 [file Image_2.TIF]

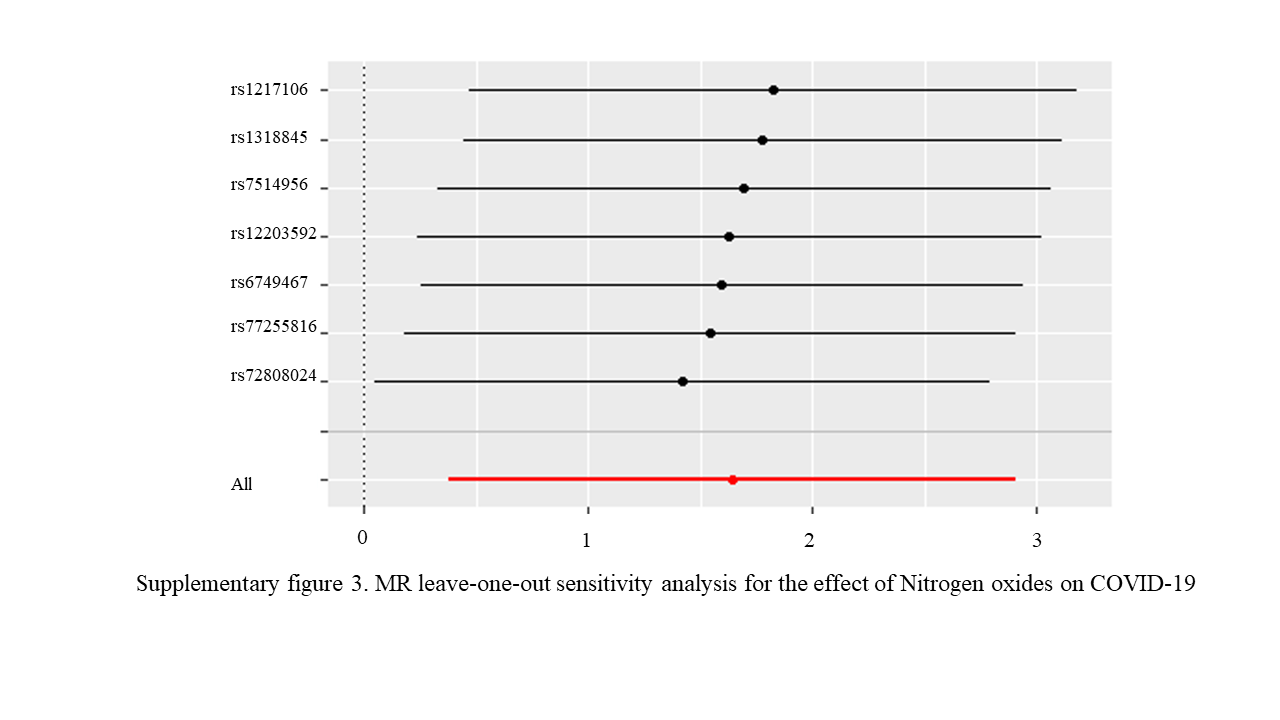

Supplement: Supplementary file 4 [file Image_3.TIF]
